# Influence of *Astragalus extract* on Gut Microbiome Regulation and Ammonia Emission Mitigation in Laying Hens

**DOI:** 10.3390/ani15050620

**Published:** 2025-02-20

**Authors:** Shasha Xiao, Kunxian Feng, Shikai Li, Miao Li, Xiliang Yan, Yinbao Wu, Jiandui Mi, Xindi Liao, Yan Wang

**Affiliations:** 1 Heyuan Branch, Guangdong Laboratory for Lingnan Modern Agriculture, College of Animal Science, South China Agricultural University, Guangzhou 510642, China; 17727616099@163.com (S.X.); 13422174008@163.com (K.F.); lishikai1028@163.com (S.L.); 15521277019@163.com (M.L.); yanxiliang1991@163.com (X.Y.); wuyinbao@scau.edu.cn (Y.W.); mijiandui@163.com (J.M.); xdliao@scau.edu.cn (X.L.); 2 Guangdong Provincial Key Lab of Agro-Animal Genomics and Molecular Breeding, South China Agricultural University, Guangzhou 510642, China; 3 National Engineering Research Center for Breeding Swine Industry, College of Animal Science, South China Agricultural University, Guangzhou 510642, China

**Keywords:** *Astragalus extract*, *Yucca extract*, laying hens, ammonia (NH_3_), gut microbiota

## Abstract

*Astragalus extract* plays a dual role in gut microbiome regulation and ammonia (NH_3_) emission mitigation in laying hens. This study explored its effects through feeding experiments, with a focus on gut microbial metabolic pathways and NH_3_ reduction mechanisms. To achieve this, both in vitro fermentation experiments and in vivo feeding trials were conducted. In the in vitro study, cecal contents from laying hens were incubated with different concentrations of AE and Yucca extract (YE) to evaluate NH_3_ production, while in the feeding trial, 58-week-old Lohmann Pink laying hens were allocated into three groups (control, 0.1% YE, and 0.1% AE) and housed in controlled-environment respiration chambers for 21 days. Measurements included NH_3_ emissions, serum biochemical indices, immune parameters, gut physicochemical properties, and 16S rRNA-based microbiota analysis. Results showed that Astragalus extract reduced NH_3_ emissions by 29.3%, achieved by lowering urease and uricase activities and promoting the conversion of ammonium nitrogen to nitrate nitrogen. Additionally, it significantly enhanced gut immune function by increasing intestinal immunoglobulin levels. Microbial community analysis revealed an increased relative abundance of *Bacteroides*, *Muribaculaceae*, and *Faecalibacterium*, which are negatively correlated with NH_3_ emissions. These microbial shifts improved ammonium nitrogen utilization via the upregulation of CTP synthase and GMP synthase activities, contributing to higher NH_3_ reduction efficiency. This study highlights *Astragalus extract* as a cost-effective and sustainable strategy to regulate gut microbiota, optimize nitrogen metabolism, and mitigate NH_3_ emissions in laying hens.

## Simple Summary

Ammonia emissions are a significant factor limiting the health and sustainable development of laying hens. Chinese herbal medicine has shown promise in reducing ammonia emissions. In our preliminary in vitro experiments, we compared the ammonia reduction effects of various Chinese herbal medicines and found that *Astragalus extract* resulted in the most significant reduction. Building on these findings, this study used feeding trials to evaluate the practical effectiveness of *Astragalus extract* in reducing ammonia emissions and to explore its underlying mechanisms. Our results showed a 29.2% reduction in ammonia emissions from laying hens. *Astragalus extract* increased the activity of CTP synthase and GMP synthase, thereby enhancing ammonia utilization efficiency. Additionally, it altered the gut microbial community structure, reduced urease and uricase activity, and consequently lowered ammonia emissions. It also significantly enhanced the hens’ immune function. Given its superior efficacy and cost-effectiveness, *Astragalus extract* holds strong potential for market application.

## 1. Introduction

Ammonia (NH_3_) is a highly irritating, colorless gas, widely recognized as the most harmful gas emitted in poultry production [1]. This gas can cause irritation to the eyes and mucous membranes of laying hens’ respiratory system [2], making them more vulnerable to respiratory diseases and adversely affecting their production performance and egg quality [3]. Workers and residents living in close proximity to livestock farms may also face potential health risks from prolonged exposure to NH_3_, such as chronic rhinitis, coughing, chest tightness, and asthma [4,5]. Furthermore, excessive NH_3_ emissions can have adverse environmental impacts, including air pollution, soil acidification, and water eutrophication [6]. Therefore, it is of great significance to seek effective measures to reduce NH_3_ emissions in the laying hen breeding environment, mitigating the harm induced by NH_3_ to laying hens, human health, and the environment.

NH_3_ in poultry is primarily produced through the cecum microbial breakdown of uric acid, undigested proteins, and amino acids [7]. Uricase and urease play a crucial role in this process, especially in an alkaline environment [8]. In the gut of poultry, *Escherichia coli* and *Candida utilis* exhibit high levels of uricase activity, enabling them to metabolize uric acid into urea [9]. *Clostridium*, *Proteus*, and *Klebsiella possess* high levels of urease activity [10]. Urease catalyzes the conversion of urea to NH_3_ at a rate that is 10 times higher than the natural rate. In addition, due to poultry’s short upper gastrointestinal tract, 20% to 30% of undigested feed components pass to the cecum for microbial fermentation, producing NH_3_ [11,12]. Thus, regulating the microbial balance and reducing uricase and urease activity in laying hens can effectively decrease NH_3_ emissions in both the gut and feces, which is crucial for controlling the concentration of NH_3_.

In recent years, in the context of green and antibiotic-free farming, Chinese herbal extracts, as feed additives and antibiotic substitutes, have received increasing attention worldwide due to their natural ingredients and widespread efficacy [13]. Chinese herbal extracts contain a rich array of active substances, including polysaccharides, saponins, alkaloids, flavonoids, and other compounds. For example, astragalus polysaccharides are the primary bioactive compounds in *Astragalus extract* (AE), contributing to its immune-modulatory and ammonia-reducing effects [14]. These substances do not require purification and can be directly applied in animals. The synergistic effects of these different substances have great potential to reduce NH_3_ emissions. Xanthine oxidase is an important enzyme in animal uric acid metabolism and can catalyze the conversion of hypoxanthine to uric acid [15]. Polysaccharides can reduce the formation of uric acid by inhibiting the activity of xanthine oxidase [16]. Moreover, saponins, alkaloids, and flavonoids can improve protein digestibility and reduce NH_3_ emissions [17]. Chinese herbal extracts such as *Yucca extract* (YE) have been proven to effectively reduce ammonia emissions [18].

*Astragalus* is one of the most popular Chinese herbal medicines, originally recorded in the “Classic of Materia Medica” during the Han Dynasty, and it has been widely used for over 2000 years [19]. It contains multiple active substances and exhibits antioxidant, antibacterial, and anti-inflammatory effects [20]. Recent research on Astragalus has primarily focused on its immunomodulatory effects and therapeutic potential in various diseases [21]. There is evidence that adding *Astragalus* to the diet can enhance the stress resistance, immunity, production performance, and antioxidant capacity of poultry [22,23]. The limited research on the effects of *Astragalus* on NH_3_ emissions in animals may hinder its promotion as a natural additive [24]. Therefore, we used YE as a positive control and conducted feeding experiments in a respiration metabolism chamber to comprehensively evaluate the impact of *Astragalus extract* on NH_3_ emissions in laying hens. In addition, gut and serum physicochemical parameters and gut microbiota composition were measured based on high-throughput sequencing to illustrate the potential mechanism underlying the effect of AE on NH_3_ emissions. The present study not only expands the repertoire of Chinese herbal extracts but also enhances our understanding of their mechanisms and applications in the poultry industry, thus providing valuable data for their future utilization in livestock production and research.

## 2. Materials and Methods

### 2.1. In Vitro Fermentation Test Analysis

Sixty 78-week-old Lohmann Pink laying hens were randomly selected and sacrificed to obtain cecal contents for the in vitro fermentation studies. The collected cecal contents were pooled and thoroughly homogenized and mixed with a buffer solution at a 1:3 ratio (weight/volume). This buffer solution contained 35 g of sodium bicarbonate (NaHCO_3_) and 4 g of ammonium bicarbonate (NH_4_HCO_3_) per liter. The resulting mixture was then filtered through four layers of surgical gauze into bottles. The bottles were placed in a 42 °C water bath, with CO₂ gas continuously bubbled through the mixture to maintain an anaerobic environment.

For the in vitro fermentation, 30 mL of the filtered inoculum and 500 mg of feed (fermentation substrate) were transferred into calibrated glass syringes (100 mL). Specific treatments were supplemented as follows: CK: no additives; YE groups: 0.01% YE (*w*/*w*), 0.1% YE (*w*/*w*); and AE groups: 0.05% AE (*w*/*w*), 0.1% AE (*w*/*w*), 0.2% AE (*w*/*w*), 0.3% AE (*w*/*w*). Six replicates were set up for each group.

After the air was evacuated from the headspace, the 72 syringes were sealed with clamps and incubated in a shaker at 42 °C and 60 rpm for 12 h. Upon the completion of the fermentation period, the syringes were placed in ice water to halt the reaction. The gas accumulated in the headspace of each syringe was collected using a 50 mL gastight syringe (Hamilton, Reno, NV, USA) and immediately transferred into a bubble absorption tube containing sulfuric acid solution to capture NH_3_.

### 2.2. Animals and Feeding

The study was conducted with sixty Lohmann Pink laying hens of similar weights (1.68 kg  ±  0.03) aged 58 weeks when the experiment was started. The hens were purchased from Peng Chang Poultry Farm Limited, Shenzhen, China. Sixty hens were randomly divided into three groups, namely, the CK group (control group) (basic diet, Table S1), YE group (diet supplemented with 0.1% YE), and AE group (diet supplemented with 0.1% AE), with 4 replicates in each group and 5 hens in each replicate. The concentration of 0.1% AE used in this feeding trial was determined based on the optimal results obtained from the in vitro fermentation study in Section 2.1. The hens were housed in respiration chambers measuring 2.0 m × 1.0 m × 1.2 m (L × W × H), providing a total floor area of 2 m^2^ per chamber. With 5 hens per chamber, the stocking density was approximately 4000 cm^2^ per hen. The feeders provided were of a stainless steel design, with dimensions of 30 cm (length) × 15 cm (width), and the nipple drinkers were installed at a height of 25 cm from the floor, with an outlet diameter of 1.5 cm. Throughout the experimental period, feed was provided to the hens twice daily, at 8 a.m. and 4 p.m., while clean drinking water was consistently available. The hens were kept in a room maintained at a temperature of 24 °C, with a 12 h light and 12 h dark schedule. The 21-day experiment consisted of 18 days of feeding and 3 days of gas collection. The average daily feed intake (ADFI), laying rate (LR), average daily egg mass (ADEM), and feed conversion ratio (FCR) of the hens were recorded and calculated during the 21-day experimental period. It is important to note that the age, weight, and feeding environment were consistent across the three groups throughout the experimental period to minimize the potential confounding effects caused by these factors.

### 2.3. Measurement of NH_3_ Emissions

NH_3_ emissions were measured with twelve respiration chambers (patent number: 201610091580.9) (Figure S1). Each chamber had a volume of 2.4 m^3^ (2 × 1 × 1.2 m) and was constructed using an enrichment cage covered with transparent poly methyl methacrylate sheets on every side except for the bottom, which was made of a steel sheet. Two doors were located in the front of each chamber, one for feeding and the other for feces removal. Drinking water was provided through a drinker nipple positioned at the front of the cage. To ensure proper gas mixing, a fan was placed above the chambers. The chambers were ventilated using an electrical pump (type: ACO-009D; Hailea, Guangzhou, China) at the outlet end, with a ventilation rate of 45 L/min monitored by a gas flow meter (type: LZB-2WB; specifications: 6–60 L/min; ZhenXing Flowtech, Yuyao, China). The gas outlet was connected to a bottle containing diluted sulfuric acid solution (300 mL of 0.25 mol/L per bottle). NH_3_ gas was collected every 2 h. The sampling process lasted for a total of 72 h, during which there was a 3 min break every 2 h to remove feces and replace the sulfuric acid solution.

### 2.4. Recovery Rate of the Respiration Chambers

The recovery rate of the system was determined following the procedure outlined by Huang [25]. Standard gas containing 1.6% NH_3_ (*v*/*v*) and 98.4% N₂ (*v*/*v*) was released from the gas bottle into the chamber through a precision reducer and flow meter to ensure a uniform release rate. The respiration chamber was ventilated, and samples of outlet air were collected at 2 h intervals, as described earlier. Once the NH_3_ concentration in the outlet air reached equilibrium, the recovery rate of the system (R) was calculated using the following formula:

R = (*M*_1_/*M*_2_) × 100% = [*M*_1_/(*M*_3_ + *M*_4_)] × 100%

where *M*_1_ is the mass of NH_3_ recovered (μg), *M*_2_ is the mass of NH_3_ release (μg), *M*_3_ is the mass of gas released by the gas bottle (μg), and *M*_4_ is the mass of gas in the inlet air (μg).

#### Measurement of NH_3_ production

NH_3_ production (μg) from the hens every 2 h was calculated as follows:

M = [(*Ms*/*R*) × 45-*Ca* × *Va*] × 100%

where M is the mass of NH_3_ produced by hens in 2 h (μg), *Ms* is the mass of NH_3_ in the absorption solution (μg), *R* is the recovery rate of the gas in the respiration chambers, *Ca* is the concentration of NH_3_ in the inlet air (μg/L), and *Va* is the volume of inlet air. The ventilation rate (45 L/min) multiplied by 2 h yielded the volume of inlet air and was corrected to standard temperature (18 °C) and pressure (1.02 kpa).

The mass of NH_3_ in solution was measured with a spectrophotometer (type: DU640; Beckman Coulter, Brea, CA, USA) based on the Chinese National Environmental Protection Standards (HJ 533-2009) [26] (determination of ammonia, Nessler’s reagent spectrophotometry).

### 2.5. Performance

The LR, ADEM, ADFI, and FCR were evaluated at the end of the experimental period.

LR (%) = total number of eggs/number of hens per chamber × 100%

ADEM (g/hen/d) = total egg weight of each group/each group total egg number

ADFI (g/d) = total amount of feed consumed per day/number of hens per chamber

FCR (g^feed^/g^egg^) = total feed consumption/total egg weight.

### 2.6. Sample Collection

At the end of the experiment, blood samples were collected from five hens in each of the four replicates per treatment, resulting in a total of 20 samples per treatment group. The samples were then centrifuged at 3500 rpm for 10 min after 30 min of standing to obtain serum samples. Throughout the experimental period, no mortalities were recorded, indicating that the treatments did not adversely affect the survival or overall health of the hens. Subsequently, all hens were euthanized by cervical dislocation. The contents of the cecum were aseptically collected and stored at −80 °C for further experiments.

### 2.7. Blood Biochemical Parameters

Serum samples from a total of 20 hens per treatment group (5 hens per chamber × 4 replicates) were used for the determination of blood biochemical parameters. Immunoglobulin A (IgA), immunoglobulin M (IgM), and immunoglobulin G (IgG) were measured by using commercial ELISA kits (Mlbio Ltd., Shanghai, China). Uric acid, urea, blood ammonia, and xanthine oxidase were measured using commercial kits (Nanjing Jiancheng Biotechnology Research Institute Ltd., Nanjing, China; kit numbers: C012-1-1, C013-1-1, A086-1-1, and A002-1-1). The pertinent details for detection were as described in the instructions of each kit.

### 2.8. Measurement of Cecal Profiles

Accurately measured 1.0 g (±0.01 g) of cecal content was mixed with 9 mL of deionized water in a 15 mL centrifuge tube. After centrifugation at 10,000× *g* for 5 min, the supernatant was collected for analysis. The concentrations of NH_4_^+^-N and NO_3_^-^-N in the cecal samples were determined using a Nessler’s reagent-based spectrophotometric method (GB/T 11894–1989) [27] and a phenol disulfonic acid-based spectrophotometric method (GB/T 7480–1987) [28], respectively. The pH of the samples was measured using a pH meter (INESA Scientific Instrument Co., Ltd., Shanghai, China). Additionally, the levels of uric acid, urea, uricase activity, and urease activity were assessed using commercial kits provided by Nanjing Jiancheng Biotechnology Research Institute Ltd., Nanjing, China (kit numbers: C012-1-1, C013-1-1, M022-1-1, and A121-1-1). Uricase activity was quantified by monitoring absorbance changes (ΔA/min) using a kinetic assay, with enzyme activity (U/mL) derived from a standard curve. The relative uricase activity for the treatment groups was calculated as a percentage of the control group (set to 100%).

### 2.9. DNA Extraction and 16S rRNA Sequencing

After the feeding experiments, DNA was extracted from the cecal contents of 60 laying hens. The bacterial DNA in the caecum content sample was extracted based on the manufacturer’s instructions for the QIAamp PowerFecal DNA Kit (Qiagen, Germany). The bacterial DNA samples that passed quality inspection were used for the 16S rRNA sequencing analysis. The hypervariable regions (V_3-4_) of the bacterial 16S rRNA gene were amplified using the appropriate primers (F: 5′-GGACTACHVGGGTWTCTAAT-3′; R: 5′-ACTCCTACGGGAGGCAGCA-3′) [29]. After amplification, the mixed PCR products were purified using a gel extraction kit (D2500, OMEGA, USA). Following the manufacturer’s recommendations, sequencing libraries were generated using the NEBNext^®^ Ultra™ IIDNA Library Prep Kit (Cat No. E7645). The library quality was evaluated on a Qubit@ 2.0 Fluorometer (Thermo Scientific) and Agilent Bioanalyzer 2100 system. Finally, the library was sequenced on an Illumina NovaSeq platform (Novogene, Beijing, China), and 250 bp paired-end reads were generated. The raw FASTQ files were merged using FLASH (v1.2.11) (VI.2.11, http://ccb.jhu.edu/software/FLASH/ accessed on 20 January 2022), and they were subsequently demultiplexed and quality-filtered using fastp (v0.20.0), On average, each sample had 65,241 clean tags. Chimeric sequences were removed using Vsearch (v2.15.0). For the obtained effective tags, denoising was performed using the DADA2 or deblur module in QIIME2 (v2021.11) software to obtain the initial amplicon sequence variants (ASVs) (default: DADA2) (https://qiime2.org/ accessed on 27 January 2022). ASVs with abundances less than 5 were filtered out. Species annotation was conducted by mapping against the Silva database (https://www.arb-silva.de/ accessed on 30 January 2022). The ASV abundance data were normalized using a standard sequence number corresponding to the sample with the fewest sequences. Microbial functions were identified using Tax4Fun, and the Kyoto Encyclopedia of Genes and Genomes (KEGG) pathway enrichment was performed by comparing the Tax4Fun results with the KEGG database [30].

### 2.10. Data Analysis

SPSS 22.0 (IBM, USA) and Microsoft Excel 2019 (Microsoft, USA) were used to perform the statistical analysis. One-way analysis of variance (ANOVA) and multiple comparisons using Duncan’s test were performed to evaluate differences between different groups. A *p* value of <0.05 was regarded as statistically significant. Bar charts and box plots were generated using Graph Prism 8.0. The Spearman correlation coefficients between the bacterial communities and NH_3_ were analyzed with SPSS 22.0, and the results were visualized with Gephi 0.9.2 software using the Network. Principal coordinate analysis (PCoA) was performed using the normalized abundance values of the bacterial communities with the vegan package in R (V4.1.2) software. Redundancy analysis (RDA) and was performed using Canoco 5.0 software.

## 3. Results

### 3.1. AE Supplementation Reduces NH_3_ Emission of Laying Hens

**Figure 1:**
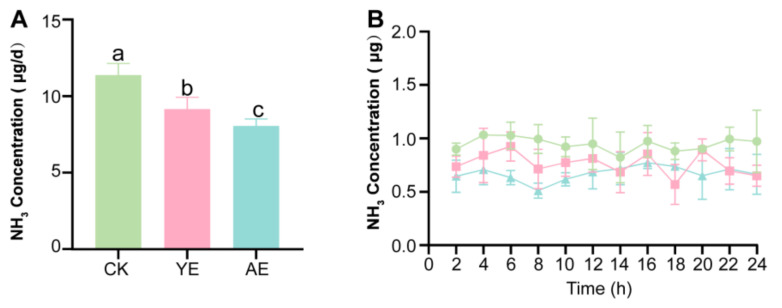
NH_3_ emissions in different treatments. (**A**) NH_3_ emissions from different treatment groups in the feeding experiment. The error bars represent the standard deviation (n = 4); the a, b, c columns with different letters are significantly different (*p* < 0.05). (**B**) Trends in NH_3_ emissions among different treatment groups in the feeding experiment.

In vitro fermentation demonstrated differential ammonia (NH_3_) mitigation across treatments (Figure 1A). Significant reductions occurred in the 0.1% YE, 0.1% AE, 0.2% AE, and 0.3% AE groups compared to the CK (*p* < 0.05), with no efficacy differences among them (*p* > 0.05). Lower concentrations (0.01% YE, 0.01% AE, 0.05% AE) showed no suppression effects (*p* > 0.05). Based on efficacy and practicality, 0.1% AE and 0.1% YE were selected for the feeding experiment as the optimal minimal-effective doses within their additive categories.

ANOVA statistical analysis indicated that NH_3_ production was significantly lower in the AE and YE groups compared to the CK group (*p* < 0.05). Additionally, we observed that the emission reduction effect of the AE group on NH_3_ was more evident than that of the YE group (*p* < 0.05) (Figure 1A). The trends in NH_3_ emissions from the laying hens are shown in Figure 1B. Ammonia emissions from the laying hens did not exhibit significant fluctuations over time. After the addition of herbal supplements, ammonia emissions were significantly reduced at all time points, indicating that the NH_3_ reduction effect of Astragalus is both sustained and stable. Furthermore, the price of AE was 75% lower than that of YE (Table S4); replacing YE with AE as a feed additive offers substantial economic benefits.

### 3.2. Effects on Production Performance and Serum Immune Parameters

Based on the obtained results, the YE group and AE group showed a tendency toward a decrease in ADFI but an increase in ADEM. However, these differences were not statistically significant compared to the CK group (*p* > 0.05) (Table S2). We speculate that this may be attributed to the short feeding time. Additionally, it is worth noting that the addition of YE significantly increases serum IgA levels (*p* < 0.05) in laying hens, and AE notably enhances the serum IgM and IgG levels of laying hens (*p* < 0.05) (Table 1). These findings indicate that both AE and YE can effectively improve the immune response of laying hens (Table 2).

### 3.3. Effects on Gut and Serum Physicochemical Indicators

**Figure 2:**
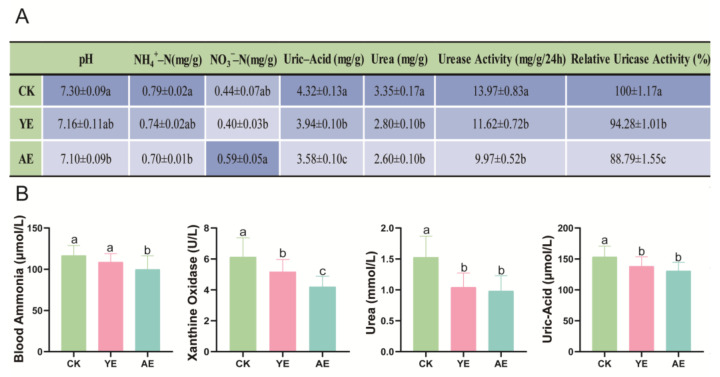
Gut and serum physicochemical indicators in different groups. (**A**) Gut physicochemical properties in different groups. The gray background indicates the average value, and the darker shades indicate higher values. (**B**) Serum physicochemical indicators in different groups. The error bars represent the standard deviation (n = 4); the a, b, c columns with different letters are significantly different (*p* < 0.05).

Uric acid is the primary form of nitrogen in the poultry gut [31]. The results of this study demonstrate that dietary supplementation with 0.1% YE or AE significantly reduced uric acid levels, urea, urease activity, and relative uricase activity (*p* < 0.05) (Figure 2A). Furthermore, the AE group exhibited a significant decrease in gut pH and NH_4_^+^-N content, as well as an increase in NO_3_^-^-N content compared to the CK and YE groups.

The concentrations of uric acid, urea, and ammonia in serum serve as important indicators of nitrogen utilization in the body [32]. Xanthine oxidase plays a key role in uric acid synthesis, and a decrease in its enzyme activity can inhibit uric acid production. In this study, the addition of AE and YE significantly reduced xanthine oxidase activity (6.14 ± 0.37 U/L, 5.17 ± 0.24 U/L, 4.20 ± 0.20 U/L, *p* < 0.05), blood uric acid levels (153.55 ± 5.14 μmol/L, 138.21 ± 4.57 μmol/L, 130.86 ± 4.07 μmol/L, *p* < 0.05), and urea levels (1.53 ± 0.10 mmol/L, 1.04 ± 0.07 mmol/L, 0.98 ± 0.07 mmol/L, *p* < 0.05) in laying hens. (Figure 2B). Additionally, the AE group exhibited significantly lower serum ammonia concentrations compared to the CK and YE groups (116.83 ± 3.95 μmol/L, 108.80 ± 3.34 μmol/L, 99.95 ± 5.38 μmol/L, *p* < 0.05). Serum ammonia concentration is greatly influenced by the ammonia concentration in the gut.

### 3.4. Effects on the Gut Microbiome Community

**Figure 3:**
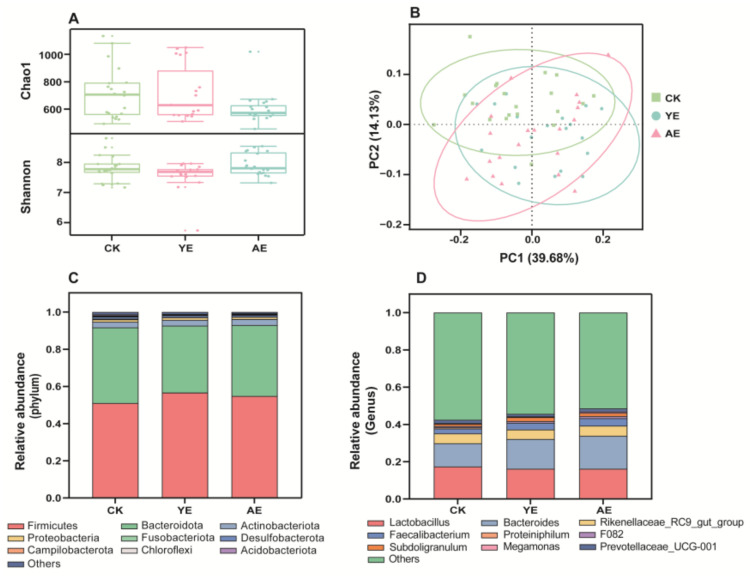
The gut microbiota community structure and diversity in different groups. (**A**) Bacterial α-diversity, (**B**) bacterial β-diversity; (**C**) bacterial community composition (phylum level); (**D**) bacterial community composition (genus level).

The effect of AE on the gut microbiome community structure was explored with 16S rRNA gene amplicon sequencing targeting the bacterial V_3-4_ hypervariable region. The alpha diversity of the microbiome is shown in Figure 3A. The Chao1 index values were as follows: CK (718.16 ± 43.63), AE (605.68 ± 25.44), and YE (719.10 ± 45.96), with no significant differences observed among the groups. For the Shannon index, the values were CK (7.81 ± 0.09), AE (7.92 ± 0.08), and YE (7.56 ± 0.11). Notably, a significant difference was detected between the AE and YE groups (*p* < 0.05), while no significant difference was observed between AE and CK. The reduction in the Chao1 index in the AE group may reflect a decrease in rare species within the gut microbiota compared to the CK group. PCoA based on the weighted UniFrac distance was used to quantify the bacterial beta diversity (Figure 3B). The results showed that compared with the CK group, the YE group or AE group changed the gut microbiome community. The stacked Kogut bar chart of the microbiome community at the phylum level is provided in Figure 3C. Firmicutes (average relative abundance: 51.1%, 56.7%, 54.8%) and Bacteroidetes (40.7%, 36.0%, 38.2%) accounted for the highest proportion in the CK, YE, and AE groups, followed by Actinobacteria (3.1%, 3.1%, 3.3%). At the genus level (Figure 3D), eleven genera had 1% relative abundance in all groups. *Lactobacillus* (17.4%, 16.1%, 16.2%), *Bacteroides* (12.4%, 16.0%, 17.7%), and *Rikenellaceae_RC9_gut_group* (5.3%, 5.1%, 5.4%) were found to be the most common in all groups. These genera are common in poultry. The differentially abundant bacteria at the genus level between the CK group and the two treatment groups are shown in Figure S2. The relative abundances of *Bacteroides*, *Faecalibacterium*, and *Muribaculaceae* were significantly higher in both the YE group and AE group than in the CK group. However, the total bacterial abundance was not affected by the different groups (Table S5). These results indicate that the AE or YE regulated some related functional microbes rather than altering the total bacterial abundance.

### 3.5. Correlation Analysis of NH_3_ Concentrations and Bacterial Genera

**Figure 4:**
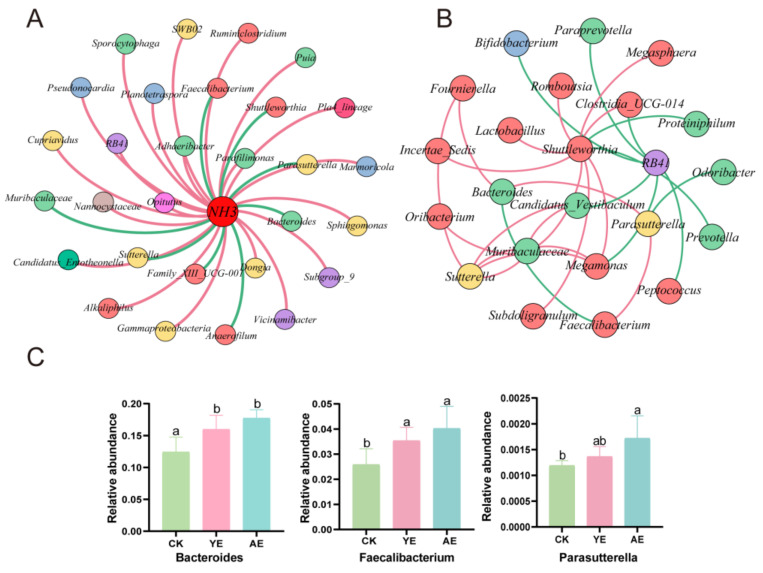
Correlation between genera and NH_3_. (**A**) Network depicting the correlations of the genus with NH_3_ emission (|r| > 0.6, *p* < 0.05). (**B**) Network depicting the correlations between bacterial genera related to NH_3_ emissions and bacterial genera with relative abundances greater than 0.1% (|r| > 0.6, *p* < 0.05). (**C**) The relative abundance of the main NH_3_-inhibiting bacteria; the a, b, c columns with different letters are significantly different (*p* < 0.05).

To investigate the relationship between NH_3_ emissions and gut bacterial genera, a network analysis was conducted using Pearson’s correlation analysis with the three groups. Among the bacterial genera, 22 showed positive correlations with NH_3_ emissions, while 8 showed negative correlations (Figure 4A, Supplemental Table S6). Notably, *RB41* exhibited a positive correlation with NH_3_ emissions but a negative correlation with other bacterial genera (Figure 4B, Supplemental Table S7). Its relative abundance decreased in the YE group and AE group compared to the CK group. Conversely, *Bacteroides*, *Parasutterella*, and *Faecalibacterium* were negatively correlated with NH_3_ emissions and *RB41* but positively correlated with *Lactobacillus* and *Shuttleworthia*. Their relative abundances significantly increased in the YE group and AE group compared to the CK group (*p* < 0.05) (Figure 4C).

### 3.6. Microbial Function Prediction Analysis

**Figure 5:**
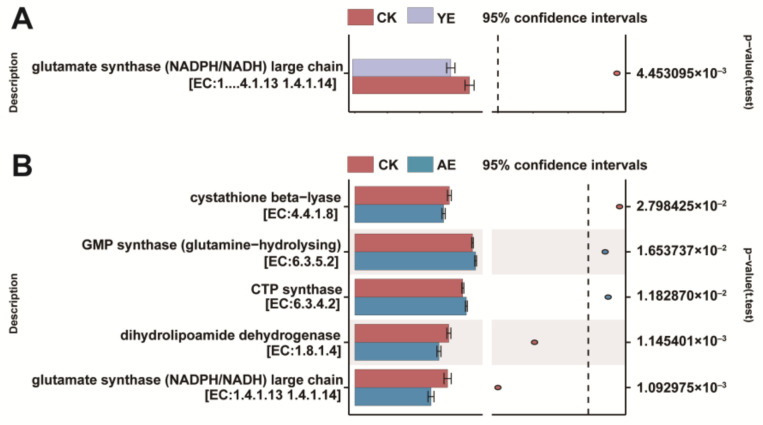
Effect of YE (**A**) or AE (**B**) on NH_3_ emission and utilization.

In addition, to explore the mechanism underlying NH_3_ emissions, Tax4Fun was employed to predict the functions of the microbiome based on 16S rRNA data. The PCoA map of the predicted KEGG gene functions was in agreement with the microbiome community structure (Figure S3). We identified the KEGG Orthology (KO) IDs that participated in NH_3_ emission and utilization in the KEGG pathway database, and the KO IDs related to NH_3_ emission were screened out of 6591 KO IDs noted in the results of this study. Glutamate synthase (EC:1.4.1.13) was significantly downregulated in both the YE group and AE group. CTP synthase (EC:6.3.4.2) and GMP synthase (EC:6.3.5.2) were significantly upregulated in the AE group compared with the CK group (*p* < 0.05) (Figure 5).

### 3.7. Factors Influencing NH_3_ Emissions

**Figure 6:**
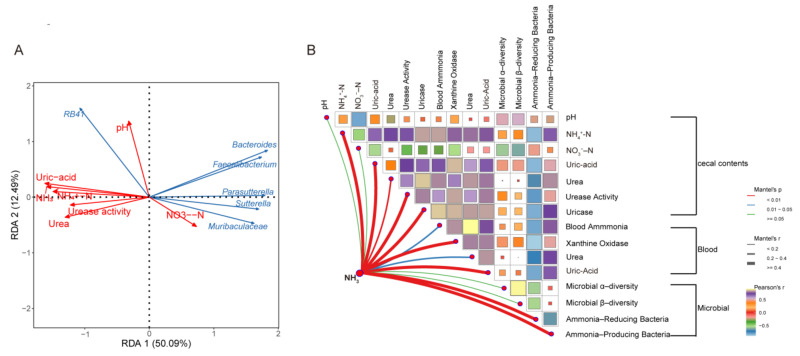
Factors influencing NH_3_ emission. (**A**) RDA showing the relationship between physicochemical properties and genus and NH_3_. (**B**) Mantel analysis of NH_3_ emission related indexes; the greater the Mantel test correlation was, the smaller the *p* value (*p* < 0.05).

Environmental factors can drive changes in the bacterial community in a complex gut environment and affect NH_3_ emission. Previous studies have shown that physicochemical properties have a significant regulatory effect on NH_3_ emission in poultry. Thus, the physicochemical properties of laying hen guts can be manipulated so that NH_3_ emission is markedly reduced in NH_3_ producers. The results of redundancy analysis (RDA) showed that uric acid and NH_4_⁺-N were the primary drivers of NH_3_ emissions (Figure 6A). The addition of AE significantly reduced the concentrations of both uric acid and NH_4_⁺-N. Further, Mantel correlation analysis revealed that ammonia-producing and ammonia-inhibiting microbes had a stronger correlation with NH_3_ emissions than microbial alpha and beta diversity (Figure 6B). This indicates a more direct relationship between specific microbial populations and NH_3_ emissions. Specifically, *Bacteroidaceae*, *Parasutterella*, and *Faecalibacterium* exhibited a negative correlation with NH_3_ emissions and a positive correlation with NO_3_⁻-N, while *RB41* showed a positive correlation with both NH_3_ emissions and pH. The addition of AE significantly reduced pH and facilitated the conversion of NH_4_⁺-N to NO_3_⁻-N, suggesting that pH may indirectly influence NH_3_ emissions. Furthermore, a strong correlation was observed between blood xanthine oxidase, uric acid, and NH_3_ emissions. Xanthine oxidase promoted NH_3_ emissions by increasing uric acid production. The addition of AE significantly reduced xanthine oxidase activity and uric acid levels. Overall, AE effectively reduced NH_3_ emissions by improving blood and gut physicochemical parameters and modulating gut microbiota.

## 4. Discussion

The management of livestock and poultry odor revolves around three fundamental strategies as follows: source reduction, process control, and end-of-pipe treatment. As the foundational approach to controlling odors, source reduction entails the inclusion of additives in the appropriate types and proportions within the feed ingredients of livestock and poultry [1]. These additives are tailored to the specific species and dietary requirements, ensuring the formulation of nutritionally balanced feed [33]. This practice promotes balancing gut microbiota in animals, enhances nutrient absorption, and mitigates the production of NH_3_. In vitro fermentation experiments showed that the addition of 0.1% YE or 0.1% AE resulted in the most effective reduction in NH_3_ emissions. This was further confirmed by feeding trials, which showed NH_3_ emission reductions of 19.53% and 29.29% for laying hens, respectively. The results indicate that AE significantly outperforms YE in reducing NH_3_ emissions from laying hens, consistent with our earlier findings from the in vitro fermentation experiments [24]. Furthermore, considering that AE is priced at 75% of the cost of YE, its use as an economically viable and effective solution for odor control becomes even more compelling. This positions AE as a promising candidate for commercial applications.

Although individual body weights were recorded at both the start and the end of the experiment, no significant differences were observed among the groups. This indicates that the experimental treatments (YE or AE supplementation) did not adversely affect the overall growth performance of the laying hens. In addition, although the differences in performance parameters (average daily feed intake, laying rate, egg mass, and feed conversion ratio) were not statistically significant between the groups, the observed trends suggest that AE supplementation may have a positive influence on feed efficiency. This could be related to improved nutrient utilization and reduced metabolic waste due to lower ammonia levels. Future long-term studies with larger sample sizes are warranted to confirm these findings and to further investigate the potential benefits of AE on production performance.

The primary source of NH_3_ in laying hens is believed to be the microbial breakdown of uric acid, undigested protein, and amino acids [7]. The process of uric acid decomposition, leading to ammonia production, involves a series of intricate chemical reactions. The key intermediate products include allantoin and urea, while uricase and urease function as rate-limiting enzymes [34]. With the addition of AE and YE, the activities of uric acid, uricase, urea, and urease enzymes in the cecum were all inhibited, indicating the suppression of uric acid decomposition by AE and YE in the cecum of laying hens. Uricase is an oxidoreductase enzyme, and its activity can be inhibited by antioxidants [35]. AE contains various bioactive substances with antioxidant properties, including alkaloids, polysaccharides, flavonoids, and saponins [36]. Urease, a large nickel-containing enzyme, can be inhibited by flavonoids and alkaloid compounds present in AE, as they can chelate with the active center of urease, resulting in the inhibition of its activity [37,38]. Furthermore, harmful bacteria such as *Prevotella* species exhibit high urease activity [39], and the addition of AE can hinder their proliferation, leading to reduced urease secretion and consequently decreasing the decomposition of uric acid and urea, thereby reducing NH_3_ emission.

The addition of AE to the diet promotes the development of the poultry’s anterior gut, increases villus height, reduces crypt depth, and enhances the digestion and absorption of intestinal proteins [40]. As a result, it decreases the entry of fermentable substances into the cecum and reduces the concentration of microbial fermentation substrates. The results of this study showed that AE significantly reduces the activity of xanthine oxidase in the blood (*p* < 0.05). Xanthine oxidase is a key enzyme involved in the synthesis of uric acid, and the reduction in its enzymatic activity indicates the inhibition of uric acid synthesis [41]. Research has shown that polysaccharides in AE can competitively bind to the binding site of xanthine oxidase with the precursors of uric acid synthesis, thereby inhibiting the synthesis of uric acid [42]. A decrease in serum ammonia, uric acid, and urea levels suggests a higher rate of protein synthesis in the body and reduced ammonia production, indicating more efficient nitrogen utilization.

When considering NH_3_ emissions from cecal fermentation, it is important to take into account the involvement of the microbiome. Our study revealed correlations between NH_3_ emissions and 30 bacterial species. Specifically, 22 bacterial species showed positive correlations with NH3 emissions, with r values ranging from 0.6 to 0.73; 8 bacterial species showed negative correlations, with r values ranging from −0.6 to −0.79. Additionally, we found that seven of these NH_3_-associated bacterial genera (six with positive correlations and one with a negative correlation) interacted with the dominant gut microbiota, which had a relative abundance greater than 0.1%. These bacteria included ammonia-reducing species such as *Bacteroides*, *Parasutterella*, *Muribaculaceae*, and *Faecalibacterium*. The addition of AE significantly increased their relative abundance. *Faecalibacterium*, known for its anti-inflammatory properties, is a commensal bacterial genus involved in reducing colitis [43]. An increase in its relative abundance can result in decreased catabolic activity towards proteins and amino acids [44]. *Bacteroides*, a probiotic used as a feed additive, primarily utilizes carbohydrates for energy and rarely utilizes nitrogen-containing organic compounds [45]. Multiple studies have demonstrated the beneficial effects of *Parasutterella* and *Muribaculaceae* on host health [46,47]. Consistent with these findings, our research also found that the addition of AE significantly enhanced the immune performance of laying hens. The significant increase in serum IgM and IgG levels observed in the AE group suggests that AE supplementation may enhance humoral immune responses. This immunomodulatory effect could be attributed to the bioactive compounds present in AE, such as flavonoids and saponins, which have been reported to modulate cytokine production and promote B-cell proliferation [48]. Collectively, these findings demonstrate that AE likely mediates the mitigation of ammonia (NH_3_) emissions and the enhancement of immune responses in laying hens through the precise modulation of key functional taxa—specifically Faecalibacterium, Bacteroides, and Muribaculaceae—without altering the overall bacterial abundance.

To explore the mechanism underlying the reduction in NH_3_ emission, Tax4Funsoftware was employed to predict the microbiome functions of the microbiome based on the 16S rRNA data [49]. Then, we identified the Kos involved in NH_3_ production and utilization in the KEGG pathways database, and we subsequently analyzed the differences in these KO IDs between the different treatment groups. We found that Glutamate synthase (EC:1.4.1.13) was significantly downregulated in both the YE group and AE group. Glutamate dehydrogenase (GDH) catalyzes the reversible oxidative deamination of L-glutamate to 2-oxoglutarate, using glutamate synthetase as a coenzyme [50]. The decreased activity of glutamate synthetase can effectively reduce NH_3_ production in this process. Additionally, CTP synthase (EC:6.3.4.2) and GMP synthase (EC:6.3.5.2) were significantly upregulated in the AE group compared to the CK group (*p* < 0.05). GMP synthase in the glutaminase domain produces ammonia, which is transferred through a channel into the amido-ligase domain, where it is ligated to UTP to form CTP in an ATP-dependent process. CTP synthase catalyzes the final, rate-limiting step of de novo CTP biosynthesis [51], and increasing GMP synthase and CTP synthase activities can increase ammonia utilization efficiency.

## 5. Conclusions

In this study, the effectiveness of AE in reducing NH_3_ emissions in laying hens was shown to be superior to that of YE. Uric acid and NH_4_^+^-N are the main physicochemical factors that promote NH_3_ emissions. *RB41* is the main bacterium that promotes NH_3_ emissions, while *Bacteroides*, *Faecalibacterium*, and *Muribaculaceae* are the main bacteria that inhibit NH_3_ emissions. The addition of AE reduced uric acid and promoted the transformation of NH_4_^+^-N to NO_3_^-^-N. The gut environment was improved by increasing the relative abundance of ammonia-inhibiting bacteria and reducing the relative abundance of ammonia-producing bacteria. In addition, AE significantly increased the immunity of laying hens, and the cost was lower than that of YE. In addition to significantly reducing ammonia emissions and enhancing immune function, AE supplementation tended to improve production performance, as indicated by favorable trends in feed conversion and egg mass. Therefore, the use of AE as a feed additive in production has good production and economic benefits.

## Figures and Tables

**Table 1:** NH_3_ emissions in different treatments in the in vitro fermentation experiment.

Group	NH_3_ Concentration (μg)
CK	23.84 ± 1.66 ^a^
0.01% YE	24.80 ± 0.57 ^a^
0.1% YE	19.10 ± 0.60 ^b^
0.01% AE	24.88 ± 1.65 ^a^
0.05% AE	23.49 ± 1.27 ^a^
0.1% AE	18.82 ± 0.39 ^b^
0.2% AE	17.26 ± 0.63 ^b^
0.3% AE	17.97 ± 0.37 ^b^

Values are expressed as the mean ± SE. Different superscripts within the same row indicate significant differences, as determined by one-way ANOVA followed by Duncan’s test (*p* < 0.05).

**Table 2:** Effects of YE and AE on serum immune parameters in laying hens.

	CK	YE	AE
IgA (mg/dL)	38.12 ± 2.85 ^b^	44.81 ± 5.56 ^a^ (*p* = 0.012)	39.83 ± 6.40 ^ab^ (*p* = 0.564)
IgM (mg/dL)	106.91 ± 4.84 ^b^	120.27 ± 3.21 ^ab^ (*p* = 0.052)	128.68 ± 6.22 ^a^ (*p* = 0.0002)
IgG (mg/dL)	385.17 ± 6.89 ^b^	408.00 ± 8.99 ^b^ (*p* = 0.079)	442.83 ± 6.50 ^a^ (*p* = 0.006)

Values are expressed as the mean ± SE. Different superscripts within the same row indicate significant differences, as determined by one-way ANOVA followed by Duncan’s test (*p* < 0.05). The *p* value indicates the significance of the difference between each treatment group and the control group.

## Data Availability

All the data generated or analyzed in this study are included in this paper. The 16S rRNA gene sequences in this study were deposited into the National Center for Biotechnology Information (NCBI) database (PRJNA967812).

## Supplementary Materials

The following supporting information can be downloaded at: https://www.mdpi.com/article/10.3390/ani15050620/s1, Table S1. Diet composition and nutrient levels; Table S2. Production performance of laying hens; Table S3. Recovery of the respiration chamber system; Table S4. Prices of YE and AE; Table S5. Abundance of the total bacteria in caecum of laying hen at different group; Table S6. Correlation between NH_3_ concentration (μg/d) and the relative abundance of the microorganism; Table S7. Correlation between NH3-associated bacterial genera and other bacterial genera with relative abundance > 0.1% (Relative abundance); Figure S1. Structure of the respiration chamber system; Figure S2. Differential analysis of genus-level bacteria; Figure S3. Principal coordinate analyses based on 16S rRNA data using Tax4Fun.
