# Supplementary material for: Influence of Astragalus extract on Gut Microbiome Regulation and Ammonia Emission Mitigation in Laying Hens
Source: Animals (Basel). 2025 Feb 20;15(5):620. doi: 10.3390/ani15050620 (PMC11898126; doi:10.3390/ani15050620)
Supplement: Supplementary file 1 [file animals-15-00620-s001.zip › animals-3439167-supplementary.pdf]

animals

Article

Influence of *Astragalus extract* on Gut Microbiome Regulation and Ammonia Emission Mitigation in Laying Hens

Shasha Xiao [1], Kunxian Feng [1], Shikai Li [1], Miao Li [1], Xiliang Yan [1,2,3], Yinbao Wu [1,2,3], Jiandui Mi [1,2,3], Xindi Liao [1,2,3] and Yan Wang [1,2,3,*]

[1] Heyuan Branch, Guangdong Laboratory for Lingnan Modern Agriculture, College of Animal Science, South China Agricultural University, Guangzhou 510642, China; 17727616099@163.com (S.X.); 13422174008@163.com (K.F.); lishikai1028@163.com (S.L.); 15521277019@163.com (M.L.); yanxiliang1991@163.com (X.Y.); wuyinbao@scau.edu.cn (Y.W.); mijiandui@163.com (J.M.); xdliao@scau.edu.cn (X.L.)

[2] Guangdong Provincial Key Lab of Agro-Animal Genomics and Molecular Breeding, South China Agricultural University, Guangzhou 510642, China

[3] National Engineering Research Center for Breeding Swine Industry, College of Animal Science, South China Agricultural University, Guangzhou 510642, China

* Correspondence: ywang@scau.edu.cn; Tel.: +86-20-85280279; Fax: +86-20-85280740

Captions:

Table S1 Diet composition and nutrient levels
Table S2 The production performance of laying hens
Table S3 The recovery of respiration chamber system
Table S4 The prices of YE and AE
Table S5 The abundance of total bacteria in caecum of laying hen at different group
Table S6 The Correlation between NH_3 and Microorganisms
Table S7 The Correlation between Bacterial related to NH_3 and Bacterial (relative abundances >0.1%)
Figure S1. The structure of respiration chamber system.
Figure S2. Differential analysis of genus-level bacteria
Figure S3. Principal coordinate analyses based on 16S rRNA data using Tax4Fun.

Academic Editor: Colin G Scanes

Received: 6 January 2025
Revised: 14 February 2025
Accepted: 18 February 2025
Published: date

Citation: Xiao, S.; Feng, K.; Li, S.; Li, M.; Yan, X.; Wu, Y.; Mi, J.; Liao, X.; Wang, Y. Influence of *Astragalus extract* on Gut Microbiome Regulation and Ammonia Emission Mitigation in Laying Hens. *Animals* **2025**, *15*, x. https://doi.org/10.3390/xxxxx

Copyright: © 2025 by the authors. Submitted for possible open access publication under the terms and conditions of the Creative Commons Attribution (CC BY) license (https://creativecommons.org/licenses/by/4.0/).

Table S1. Diet composition and nutrient levels.

Ingredients	Content（%）	Nutrient levels	Content
Maize meal	62.00	ME（MJ/kg）	11.5
Soybean meal	26.00	CP（%）	16.7
Soybean oil	1.00	Ca（%）	4.00
Limestone	8.00	TP（%）	0.45
Salt	0.30	AP（%）	0.32
DL-Met	0.16	Lys（%）	0.80
Monocalcium phosphate	1.54	Met（%）	0.38
Premix [1]	1.00		
Total	100.00		

[1]The premix provided the following per kg of diets: VA120 KIU, VD3 40 KIU, VE 15 IU, VB1 1 mg, VB2 8.5 mg VB6 8 mg, Niacin 32.5 mg, Folate 10 mg, Choline 500 mg, Cu 8 mg，Fe 60 mg，Zn 40 mg，Mn 65 mg，I 1 mg；Se 0.3 mg.

Table S2. The production performance of laying hens.

Items	CK group	YE group	AE group
ADFI（g/d）	139.17±3.57	135.00±3.49 (P=0.405)	138.33±2.92 (P=0.867)
LR（%）	88.33±5.51	86.67±4.30 (P=0.806)	86.67±3.60 (P=0.805)
ADEM (g/hen/d)	64.90±0.82	66.79±0.36 (P=0.104)	66.47±0.65 (P=0.167)
FCR (g^{feed}/g^{egg})	2.14±0.07	2.02±0.05 (P=0.206)	2.08±0.05 (P=0.265)

Values are expressed as mean ± SE. Different superscripts within the same row indicate significant differences as determined by one-way ANOVA followed by Duncan's test ($p < 0.05$). The p indicates the significance of the difference between each treatment group and the control group.

Table S3. The recovery of respiration chamber system.

Chamber number	Recovery（%）
1	82.64±3.37
2	82.69±3.72
3	90.01±3.31
4	93.24±4.08
5	86.73±2.05
6	80.45±3.07
7	82.48±1.62
8	91.54±4.7
9	96.71±1.46
10	86.55±1.93
11	85.96±5.07
12	95.12±2.77

Table S4. The prices of Yucca extract and Astragalus extract.

Group	Prices(USD/Kg)
Astragalus extract	41.46
Yucca extract	54.88

Table S5. The abundance of total bacteria in caecum of laying hen at dif ferent group.

Group	Log (copies/g)
CK	4.16±0.15
YE	3.95±0.21
AE	4.27±0.09

Table S6. The Correlation between NH_3 concentration (μg/d) and and the relative abundance of the microorganism

Bacterial genera	P-value	Correlation
Bacteroides	0.002223	-0.7902098
Marmoricola	0.006538	0.73431588
Puia	0.00757	0.72551886
SWB02	0.014201	0.6838129
Pseudonocardia	0.014753	0.68105785
Sphingomonas	0.016569	0.67250541
Gammaproteobacteria	0.022009	0.65044364
Adhaeribacter	0.022009	0.65044364
Alkaliphilus	0.022009	0.65044364
Candidatus_Entotheonella	0.022009	0.65044364
Subgroup_9	0.022009	0.65044364
Sporocytophaga	0.025084	0.6396925
Parafilimonas	0.025084	0.6396925
Planotetraspora	0.025084	0.6396925
Nannocystaceae	0.025084	0.6396925
Opitutus	0.025084	0.6396925
Pla4_lineage	0.025084	0.6396925
Vicinamibacter	0.029419	0.62605706
Cupriavidus	0.030997	0.6214537
Ruminiclostridium	0.033315	0.61498955
RB41	0.035395	0.60945803
Dongia	0.03688	0.60564882
Anaerofilum	0.038588	-0.6013986
Family_XIII_UCG-001	0.038159	-0.6024528
Sutterella	0.035806	-0.6083916
Muribaculaceae	0.030676	-0.6223776
Shuttleworthia	0.030676	-0.6223776
Parasutterella	0.010245	-0.7062937
Faecalibacterium	0.005801	-0.7412587

Table S7. The Correlation between NH_3-associated bacterial genera and other bacterial genera with relative abundance >0.1% (Relative abundance)

NH_3-associated bacterial genera	Bacterial (relative abundances >0.1%)	P-value	Correlation
Shuttleworthia	*Prevotella*	0.001832	-0.7988082
Bacteroides	*Fournierella*	0.043778	-0.7762238
Parasutterella	*Bacteroides*	0.003446	-0.7692308
Bacteroides	*Parasutterella*	0.047218	-0.7692308
RB41	*Candidatus_Vestibaculum*	0.020794	-0.6549922
Faecalibacterium	*Bacteroides*	0.022034	-0.6503497
Parasutterella	*Odoribacter*	0.026097	-0.6363636
RB41	*Clostridia_UCG-014*	0.029113	-0.6269712
RB41	*Paraprevotella*	0.03152	-0.6199659
RB41	*Bifidobacterium*	0.039598	-0.5989501

RB41	*Peptococcus*	0.04259	-0.5919449
Shuttleworthia	*Proteiniphilum*	0.042868	-0.5913124
RB41	*Megamonas*	0.047374	-0.581437
Shuttleworthia	*Subdoligranulum*	0.038588	0.6013986
Shuttleworthia	*Muribaculaceae*	0.03317	0.61538462
Sutterella	*Megamonas*	0.03317	0.61538462
Muribaculaceae	*Megamonas*	0.02832	0.62937063
Shuttleworthia	*Lactobacillus*	0.026097	0.63636364
Sutterella	*Fournierella*	0.022034	0.65034965
Sutterella	*Candidatus_Vestibaculum*	0.022034	0.65034965
Parasutterella	*Faecalibacterium*	0.012593	0.69230769
Shuttleworthia	*Romboutsia*	0.005801	0.74125874
Shuttleworthia	*Clostridia_UCG-014*	0.005124	0.74825175
Shuttleworthia	*Incertae_Sedis*	0.043778	0.77622378
Muribaculaceae	*Oribacterium*	0.027611	0.8041958
Shuttleworthia	*Megasphaera*	0.008734	0.86013986
Muribaculaceae	*Candidatus_Vestibaculum*	0.006993	0.86713287
Sutterella	Muribaculaceae	0.000114	0.88811189
Muribaculaceae	*Sutterella*	0.003326	0.88811189
Shuttleworthia	*Megamonas*	8.37E-05	0.8951049

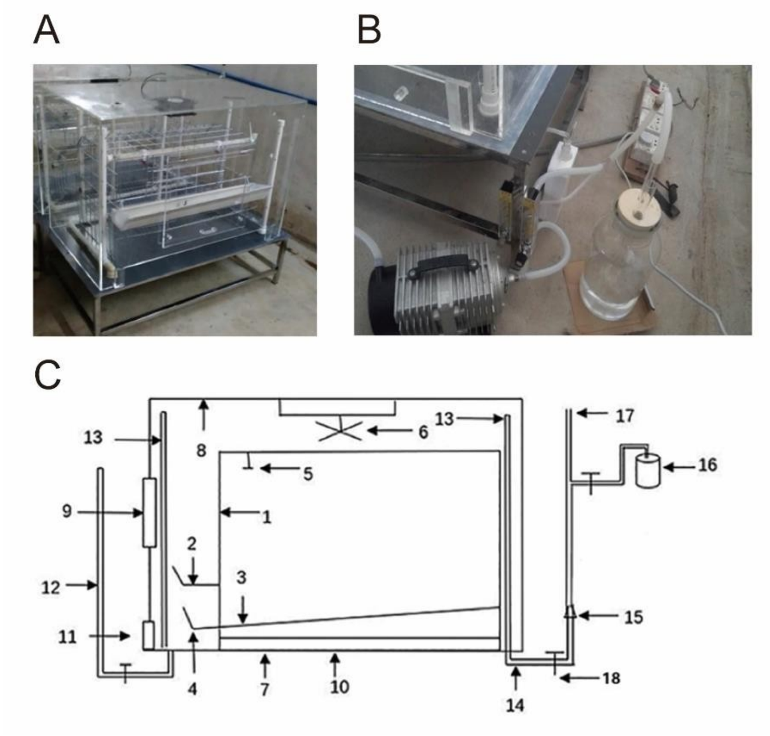

Figure S1. The structure of respiration chamber system. A The physical picture of respiration chamber. B Absorption bottle for gas collection. C The sketch of respiration chamber. 1 The inner cage, Feed trough, 3 Inner floor, 4 Eggs trough, 5 Drinker, 6 fan, 7 PVC floor, 8 Organic glass cover, 9 Baffle of feeding, 10 Feces collection, 11 Baffle of feces collection, 12 Air inlet, 13 Air outlet, 14 Total air outlet, 15 Air pump, 16 Ammonia absorption bottle, 17 Exhaust hose, 18 Hydrogen sulfide ab- sorption bottle, 19 Flowmeter.

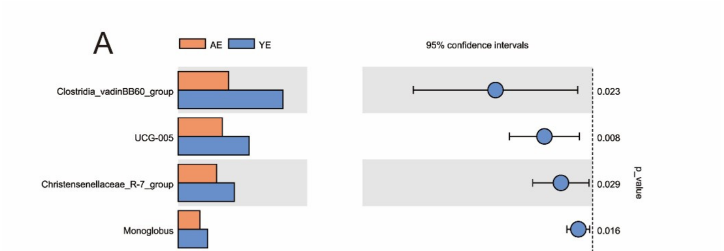

Figure S2. Differential analysis of genus-level bacteria between the AE group and YE group (A), the AE group and CK group (B), and the YE group and CK group (C).

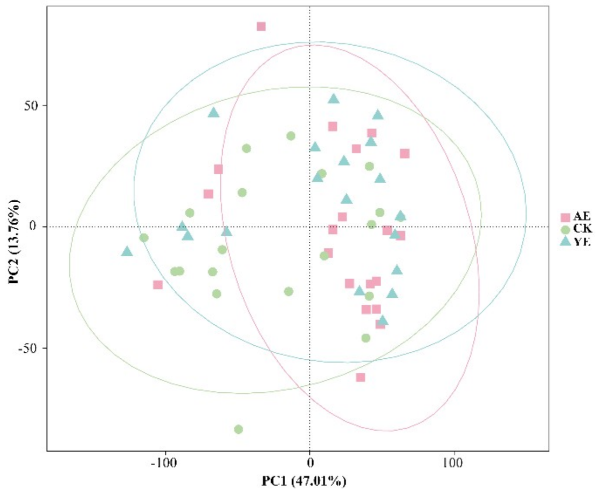

Figure S3. Principal coordinate analyses based on 16S rRNA data using Tax4Fun.CK-ControGroup, AE-Astragalus extract, YE- Yucca extract.
